# Asymmetric Dimethylation of Ribosomal S6 Kinase 2 Regulates Its Cellular Localisation and Pro-Survival Function

**DOI:** 10.3390/ijms24108806

**Published:** 2023-05-15

**Authors:** Mahmoud I. Khalil, Heba M. Ismail, Ganna Panasyuk, Anna Bdzhola, Valeriy Filonenko, Ivan Gout, Olivier E. Pardo

**Affiliations:** 1 Molecular Biology Unit, Department of Zoology, Faculty of Science, Alexandria University, Alexandria 21568, Egypt; 2 Department of Biological Sciences, Faculty of Science, Beirut Arab University, Beirut P.O. Box 11-5020, Lebanon; 3 Department of Infection, Immunity and Cardiovascular Disease, University of Sheffield, Sheffield S10 2TN, UK; h.ismail@sheffield.ac.uk; 4 Healthy Lifespan Institute (HELSI), University of Sheffield, Sheffield S10 2TN, UK; 5 Institut Necker-Enfants Malades (INEM), 75015 Paris, France; ganna.panasyuk@inserm.fr; 6 INSERM U1151/CNRS UMR 8253, Université de Paris Cité, 75015 Paris, France; 7 Department of Cell Signaling, Institute of Molecular Biology and Genetics, National Academy of Sciences of Ukraine, 03143 Kyiv, Ukrainefilonenko@imbg.org.ua (V.F.); i.gout@ucl.ac.uk (I.G.); 8 Department of Structural and Molecular Biology, University College London, London WC1E 6BT, UK; 9 Institute of Healthy Ageing, University College London, London WC1E 6BT, UK; 10 Division of Cancer, Department of Surgery & Cancer, Faculty of Medicine, Imperial College London, London W12 0NN, UK

**Keywords:** arginine methylation, serine/threonine kinases, AT-hook, methyltransferases, SCLC

## Abstract

Ribosomal S6 kinases (S6Ks) are critical regulators of cell growth, homeostasis, and survival, with dysregulation of these kinases found to be associated with various malignancies. While S6K1 has been extensively studied, S6K2 has been neglected despite its clear involvement in cancer progression. Protein arginine methylation is a widespread post-translational modification regulating many biological processes in mammalian cells. Here, we report that p54-S6K2 is asymmetrically dimethylated at Arg-475 and Arg-477, two residues conserved amongst mammalian S6K2s and several AT-hook-containing proteins. We demonstrate that this methylation event results from the association of S6K2 with the methyltransferases PRMT1, PRMT3, and PRMT6 in vitro and in vivo and leads to nuclear the localisation of S6K2 that is essential to the pro-survival effects of this kinase to starvation-induced cell death. Taken together, our findings highlight a novel post-translational modification regulating the function of p54-S6K2 that may be particularly relevant to cancer progression where general Arg-methylation is often elevated.

## 1. Introduction

Ribosomal protein S6 kinases (S6Ks) are serine/threonine kinases which are downstream effectors of the extracellularly regulated kinases 1/2 (ERK1/2), the phosphoinositide 3-kinase (PI3K), and the mammalian target of rapamycin (mTOR) [1]. Despite their sequence similarity, S6K1 and 2 are involved in distinct cellular processes and many studies have shown different regulatory mechanisms for S6K2 [2,3,4,5]. S6Ks are encoded by two genes: *RPS6KB1-* and *RPS6KB2*-encoding S6K1 (p85-, p70-, and oncogenic p31-S6K1 isoforms [1]) and S6K2 (p56- and p54-S6K2 isoforms). The longer isoforms of S6K1 and S6K2 (p85-S6K1 and p56-S6K2) contain 23 and 13 amino acid N-terminal extensions, respectively, that are absent in the shorter isoforms and contain a nuclear localisation signal (NLS). S6K2 has an additional NLS and a proline-rich domain in its C-terminal domain [6]. S6K2 interacts with multiple proteins in the nucleus [3] and its nuclear localisation provides it with a distinct role from S6K1 in cell division and transcriptional regulation [3]. S6K2 has increased nuclear localisation in human breast cancer [7] and has been linked to pro-survival and chemoresistance signalling in various cancers through the phosphorylation of the nuclear RNA-binding protein hnRNPA1 [2]. In addition, experiments in HeLa and NIH3T3 cells showed that S6K2 is active throughout the cell cycle and although there are no changes in S6K2 expression levels in different cell cycle stages, S6K2 exhibited slightly higher activity in the G2 and M phases. The roles of S6K2 in the G2 and M phases are not fully understood [8].

Protein arginine methylation is a post-translational modification (PTM) that is as common, although not as dynamic, as phosphorylation, acetylation, and ubiquitination in mammals where it controls various biological processes [9]. Protein arginine methyltransferases (PRMTs) catalyse the transfer of a methyl group from S-adenosylmethionine (SAM/AdoMet) to arginine within polypeptides. Methylated arginines have lower hydrogen bonding capacity and increased hydrophobicity, thereby modulating interactions with other proteins or nucleic acids [9]. In particular, proteins with certain domains (i.e., Tudor, PHD, and WD40) can selectively bind to methylated arginine. Nine PRMTs, with relatively conserved catalytic core domains, are classified into three classes (type I, type II, and type III) according to their catalytic activity and mode of methylation [9]. Type I comprises six PRMTs (PRMT1, -2, -3, -4, -6, and -8) and type II two (PRMT5 and -9). PRMT7 is the only type III PRMT and catalyses only monomethylation (MMA) [9]. Although PRMT1 is the predominant member, the lack of any PRMT cannot be compensated by others [10,11]. PRMTs promote arginine asymmetric and symmetric dimethylation (aDMA and sDMA, respectively), or MMA, although the functional consequences of sDMA versus aDMA are uncertain [9].

Most proteins targeted by PRMTs possess glycine–arginine rich (GAR), RGG, RG repeats, or RXR sequence motifs [9]. Since we demonstrated that the S6K2 C-terminus contains two overlapping RXR motifs (RXRXR), within its nuclear localisation signal (NLS), previously shown to be targeted by PRMTs in other substrates [9], we therefore hypothesised that these RXR motifs may be arginine-methylated and regulate the biological function of this kinase.

Here we demonstrate that p54-S6K2 is asymmetrically dimethylated on Arg-475/477. We show that PRMT1, 3, and 6 bind and methylate S6K2, promoting its nuclear localisation and rescue from starvation-induced cell death. Hence, we provide the first evidence that aDMA regulates the biological functions of p54-S6K2.

## 2. Results and Discussion

S6K2 is mapped to the chromosomal 11q13 locus, commonly amplified in various cancers [12] where it was shown to regulate cell survival [2,13,14]. We describe for the first time the aDMA of S6K2 and the role of this modification in cell survival. Unlike phosphorylation, acetylation, and ubiquitination which have been extensively examined, arginine methylation has been less investigated.

### 2.1. S6K2 Interacts with PRMT1, PRMT3, and PRMT6 In Vivo and In Vitro

**Figure 1:**
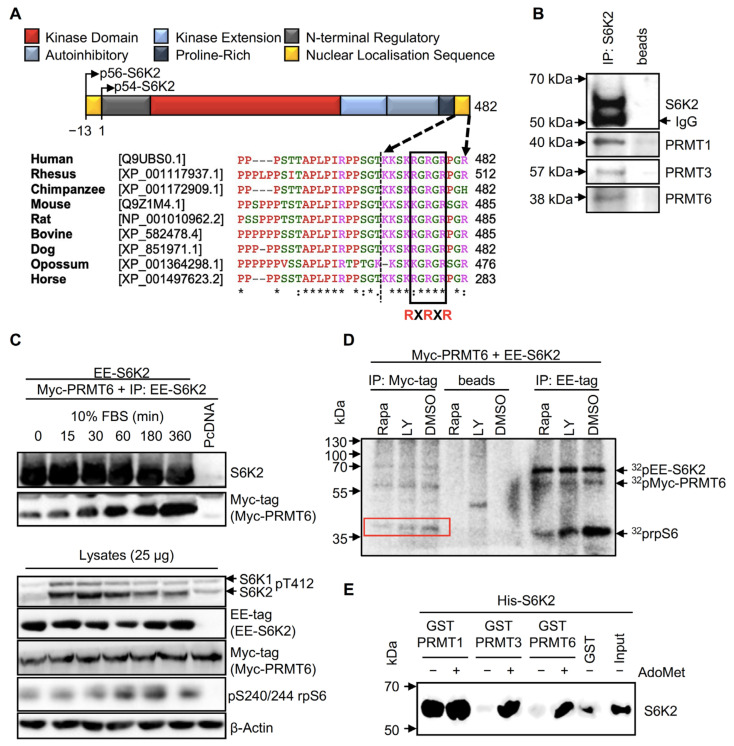
S6K2 interacts with PRMT1, 3, and 6 in vivo and in vitro. (**A**) Protein domain organisation of S6K2 and alignment of the amino acid sequences in its extreme C-terminus. The conserved Arginine-X-Arginine motif (RXRXR) is boxed, and “X” represents any amino acid. An “*” represents positions which have a single, fully conserved residue. (**B**) Endogenous S6K2 interacts with PRMTs (1, 3, and 6) in cells. S6K2 was immunoprecipitated from HEK293 and interacting proteins were detected by immunoblotting. Protein A-Sepharose beads (beads) alone were used as a control. (**C**) The interaction between S6K2 and PRMT6 is induced by serum stimulation. HEK293 cells expressing Myc-PRMT6 and EE-S6K2 or pcDNA3.1 empty vector (pcDNA) were incubated in serum-free media for 24 h prior to treatment with 10% FBS for the indicated times. The lysates were analysed directly or immunoprecipitated with an anti-EE-tag antibody prior to SDS-PAGE/immunoblotting with the indicated primary antibodies. (**D**) S6K2 activity is immunoprecipitated with PRMT6. Lysates from HEK293 cells expressing Myc-PRMT6 and EE-S6K2 were immunoprecipitated with either anti-EE-tag or anti-Myc-tag antibodies following treatment for 30 min with 100 nM Rapamycin (Rapa), 50 µM LY294002 (Ly), or DMSO (vehicle). The immune complexes were subjected to in vitro kinase assay using 80S ribosomes as substrate in the presence of [γ-32P]ATP prior to SDS-PAGE and autoradiography. The phosphorylated ribosomal S6 proteins (rpS6) were phosphorylated with the immunoprecipitated Myc-PRMT6 (red-boxed). Control IP was achieved by incubating the lysates with protein A-Sepharose beads (beads). (**E**) PRMT1, PRMT3, and PRMT6 interact with S6K2 in vitro. GST pull-down assays were performed using His-S6K2 with GST, GST-PRMT1, GST-PRMT3, or GST-PRMT6 in the presence or absence of 200 μM AdoMet, followed by immunoblotting with anti-S6K2 antibody. The input is 0.5 μg His-S6K2. (**B**–**E**) All data shown are representative of a minimum of *n* = 3 biological repeats.

The extreme C-terminus of human S6K2 shows significant sequence conservation across mammalian species (Figure 1A), including two overlapping RXR motifs (RXRXR). As similar motifs in other substrates have previously been shown to be targeted by PRMTs [9], we hypothesised that these residues in S6K2 may be arginine-methylated by these enzymes.

Given that interaction of PRMTs with their substrates is required for methylation, we performed co-immunoprecipitation experiments in HEK293 cells which revealed that endogenous S6K2 co-immunoprecipitated with PRMT1, 3, and 6 (Figure 1B). Furthermore, in HEK293 cells overexpressing Myc-tagged PRMT6 and EE-tagged S6K2, this interaction was rapidly (30 min) induced by serum stimulation and steadily increased over 6 h (Figure 1C). However, kinase activation of S6K2 is not required for this interaction as inhibition of mTOR by Rapamycin or of PI3K by LY294002, both abolishing S6 kinase activity as judged from rpS6 phosphorylation, did not prevent the association of S6K2 with PRMT6 (Figure 1D).

To test whether PRMT-S6K2 interaction is direct and whether it is impacted by the presence of the methyl donor, His-S6K2 was incubated with GST-PRMT1, 3, or 6 bound to glutathione-Sepharose beads in the presence or absence of the methyl donor, AdoMet. Figure 1E shows that AdoMet promotes the interaction of S6K2 with PRMT3 and 6, suggesting that the activity or conformational changes of these PRMTs may regulate their binding. In contrast, binding to PRMT1 is constitutive, similar to the previously reported PRMT1/hnRNP interactions [15]. In addition, while methylation leaves the overall charge of arginine unchanged, it increases steric hindrance which may disrupt hydrogen bonding [10], modulating protein–protein interactions [16]. S6K2 and PRMTs may regulate each other’s activity through binding, a possibility supported by the prior literature suggesting that interactors can either inhibit or activate the methyltransferase activity of PRMTs [17,18,19,20].

### 2.2. S6K2 Is Methylated In Vitro and in Cells

**Figure 2:**
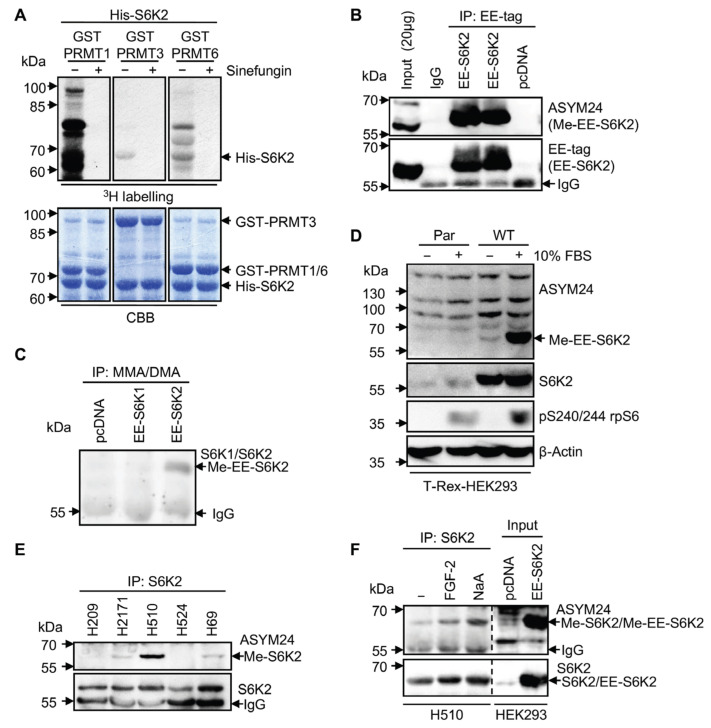
S6K2 is methylated in vitro and in cells. (**A**) In vitro methylation of recombinant S6K2. In vitro methylation assays with His-S6K2 and GST-PRMTs (1, 3, or 6) in the presence of [^3^H]AdoMet, with or without Sinefungin (500 μM). Total amounts of His-S6K2 and GST-PRMTs are shown by Coomassie staining (CBB). (**B**) Transiently overexpressed S6K2 is methylated in HEK293 cells. Lysates from HEK293 cells expressing EE-S6K2 or pcDNA3.1 empty vector (pcDNA) were immunoprecipitated with an anti-ASYM24 or anti-EE-tag antibody. IgG was used as an immunoprecipitation control. Input: total lysates from HEK293 cells overexpressing EE-S6K2. (**C**) Methylated EE-S6K2 is immunoprecipitated with an MMA/DMA antibody in T-Rex-HEK293 cells. Lysates from cells overexpressing EE-S6K1, EE-S6K2, or the pcDNA3.1 empty vector (pcDNA) were immunoprecipitated with an anti-Mono/DiMethyl Arginine (MMA/DMA) antibody, followed by immunoblotting with an anti-S6K1 and anti-S6K2 mixture of primary antibodies. (**D**) S6K2 methylation is induced by serum stimulation. T-Rex-HEK293 cells overexpressing pcDNA3.1 empty vector (Par) or EE-S6K2 (WT) were induced with 1 µg/mL tetracycline for 24 h. The cells were starved in serum-free media (−) for 24 h or stimulated with 10% FBS for 1 h (+) following starvation. The lysates were analysed by SDS-PAGE and immunoblotted with the indicated primary antibodies. (**E**) S6K2 is methylated in SCLC cell lines. Lysates from the indicated SCLC cell lines were subjected to immunoprecipitation with an anti-S6K2 antibody, followed by immunoblotting with an anti-ASYM24 or anti-S6K2 antibody. (**F**) S6K2 methylation in H510 cells is induced by FGF2 and sodium arsenite. The lysates from untreated (−), 0.1 ng/mL FGF-2-treated (5 min), or 1 mM sodium arsenite (NaA)-treated (30 min) H510 cells were subjected to immunoprecipitation with an anti-S6K2 antibody, followed by immunoblotting with an anti-ASYM24 or anti-S6K2 antibody. Input: total lysates from HEK293 cells overexpressing EE-S6K2 or the pcDNA3.1 empty vector (pcDNA). (**A**–**F**) All data shown are representative of *n* = 3 biological repeats.

Given the direct interaction between S6K2 and PRMTs, we next tested if S6K2 is a substrate of PRMTs. We performed in vitro methylation assays which revealed that full-length S6K2 can be methylated by PRMT1, 3, and 6 (Figure 2A). This was prevented by the SAM-dependent methyltransferase inhibitor, Sinefungin, a competing analogue of AdoMet [9].

All PRMTs tested here are type I enzymes catalysing the formation of asymmetric dimethylarginine (aDMA). To assess whether S6K2 was modified in the cells, we used ASYM24, an antibody that specifically recognises aDMA [21]. Ectopically expressed EE-S6K2 immunoprecipitated from HEK293 cells was detected by ASYM24, confirming that aDMA occurred in vivo (Figure 2B). This was confirmed by the detection of EE-S6K2 in MMA/DMA immunoprecipitates from HEK293 cells (Figure 2C). However, this modification was not detected in ectopically expressed EE-S6K1, showing that this PTM is specific for S6K2, possibly due to amino acid sequence differences in their C-terminus (KSKRGRGRPGR).

As binding of S6K2 to PRMT6 is serum-inducible, we investigated whether S6K2 methylation could be similarly regulated. Transiently expressed EE-S6K2 from HEK293 cells showed that S6K2 methylation was strongly induced following serum stimulation (Figure 2D). The dynamic nature of S6K2 methylation suggests the existence of a negative regulator for this PTM, such as a specific demethylase. Three scenarios for S6K2 demethylation can be proposed to be tested in future work: (1) enzyme-catalysed demethylation [22]; (2) the conversion of MMA to citrulline by arginine deiminases preventing subsequent methylation [23]; and (3) the replacement of the arginine-methylated pool by unmodified newly synthesised protein [24].

aDMA-containing proteins have been suggested to play an essential role in oncogenesis, with them being highly elevated in immortalised cell lines [10]. We previously reported that S6K2, but not S6K1, mediated the survival of small-cell lung cancer (SCLC) cells [2]. As S6K2, but not S6K1, is arginine methylated in our experiments, we wondered if this could be correlated with its pro-survival functions. To test it, we analysed the methylation levels of endogenous S6K2 immunoprecipitated from a panel of SCLC cell lines and found that H2171, H69, and H510 cells showed S6K2 methylation, with the signal in the H510 cells being particularly strong (Figure 2E). aDMA was induced in H510 cells treated with FGF-2, which promotes survival in this cell line through S6K2 activation [2], or sodium arsenite, an activator of S6Ks (Figure 2F). Therefore, our results suggest that S6K2 aDMA modification is dynamic. Further testing to identify stimuli-inducing or not S6K2 arginine methylation would help understand how widely this PTM participates in signalling specificity.

The interaction of S6K2 with its partners may be regulated by aDMA. Indeed, the two overlapping RXR motifs of S6K2 are preceded by a proline-rich region (PRR), a domain type involved in protein–protein interaction. Interestingly, arginine methylation regulates the binding of PRR to WW and SH3-domain-containing proteins. For instance, hnRNPK arginine methylation near its three PRRs diminishes its association with SRC kinase, reducing its activation and tyrosine phosphorylation of hnRNPK [25]. Similarly, aDMA within the PRR of the RNA-binding protein Sam68 modulates its interaction with SRC [26]. It is possible that SRC-mediated phosphorylation of S6K2 in vivo [27] could be affected by arginine methylation. Systematic identification of S6K2 interactors through its PRR would provide a deeper understanding of how methylation modulates its function.

### 2.3. Methylation of S6K2 Occurs at Arg-475 and Arg-477

**Figure 3:**
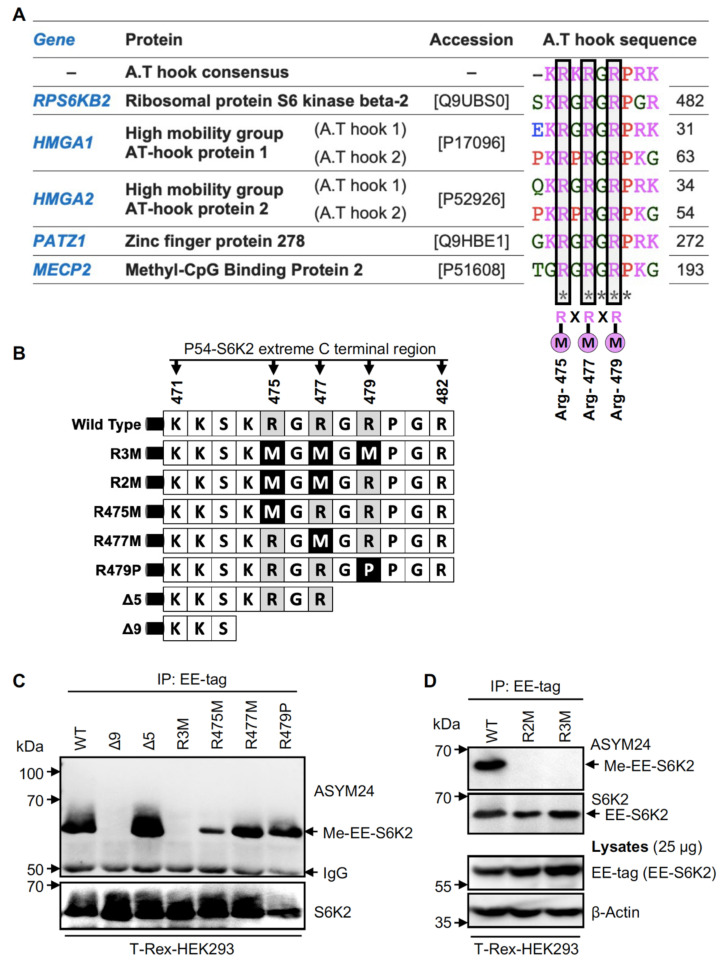
S6K2 is methylated at Arg-475 and Arg-477. (**A**) Sequence alignment of the extreme C-terminal region of S6K2 and selected AT-hook-containing proteins. The putative arginine methylation sites are boxed. RXRXR; consensus sequence for PRMTs substrates. “X” is any amino acid. An “*” represents positions which have a single, fully conserved residue. (**B**) Schematic representation of S6K2 mutants. Single, double, or triple substitution mutants (Arg to Met or Pro) are generated in addition to two truncated mutants (Δ5 and Δ9). (**C**,**D**) S6K2 is dimethylated at Arg-475/477. Lysates from T-Rex-HEK293 cells stably expressing EE-S6K2 wild type (WT), or different mutants were subjected to immunoprecipitation with an anti-EE-tag antibody followed by immunoblotting with the indicated antibodies. (**D**) Total lysates were used as a control. All data shown are representative of *n* = 3 biological repeats.

Most PRMTs preferentially methylate substrates within clusters of RGG⁄RXR box motifs [9] and within glycine–arginine-rich (GAR) motifs, present in many DNA- and RNA-binding proteins [28]. While S6K2 contains no GAR motif, it has two overlapping RXR motifs at its extreme C-terminus (RGRGR) encompassing R475, R477, and R479. Interestingly, these three residues are highly conserved in the AT-hook domains of some DNA-binding proteins, including the high mobility group (HMG) proteins (Figure 3A). Interestingly, PRMT6 dimethylates HMGA1a on Arg-57/59 in the second and Arg-83/85 in the third AT-hook motif [29], sites equivalent to those identified here for S6K2.

To determine the role of this RXR motif in the dimethylation of S6K2, we mutated Arg-475, Arg-477, and Arg-479 individually and in combination (R475M, R477M, R479P, R475M/R477M (R2M), and R475M/R477M/R479M (R3M)) (Figure 3B). We also truncated the last 5 or 9 amino acids from the C-terminus (Δ5 and Δ9, respectively). Overexpression in HEK293 cells showed that the Δ5 mutant was methylated similarly to the wild-type protein, while Δ9 truncation abolished methylation (Figure 3C). This narrowed down the methylation sites to R475 and R477. Notably, all single-point mutants were less methylated than the wild-type or Δ5 mutant, suggesting that R479 still modulates this PTM, maybe through stabilising the interaction with PRMTs. However, the double (R2M) and triple (R3M) mutants both fully prevented the methylation of S6K2, suggesting R475 and R477 as the two major sites modified (Figure 3D).

AT-hook motifs are non-classical DNA-binding domains that interact with short stretches of adenines and thymidines (4–6 bp) centred on the sequence AA(T/A)T in the DNA minor groove. The presence of an AT-hook motif in S6K2 suggests that it may bind DNA, and dimethylation might modulate the affinity or sequence specificity of this binding to impact the biological output. Indeed, this is observed for other PRMT targets that harbour nucleotide-binding activity [16]. For instance, the efficiency of PRMT1, 3, and 6 to methylate HMGA1 proteins is markedly reduced upon binding of AT-rich regions to DNA [30]. It is suggested that DNA may physically hinder the accessibility of the AT-hooks to PRMTs due to the rigid conformation of HMGA1 induced by DNA binding. Other studies reported that arginine methylation enhanced the DNA-binding capacity of PRMT substrates. For instance, methylation of DNA polymerase β by PRMT6 increases its DNA binding capacity [31]. Conversely, methylated hnRNPA1 exhibits decreased DNA affinity compared with its unmethylated form [32,33]. Likewise, methylation of signal transducer and activator of transcription 1 (STAT1) decreases its binding to DNA and subsequent function [34,35]. Hence, the potential for S6K2 to bind DNA and how dimethylation may regulate this should be formally investigated.

### 2.4. S6K2 Methylation Modulates Its Subcellular Localisation and Pro-Survival Effects

**Figure 4:**
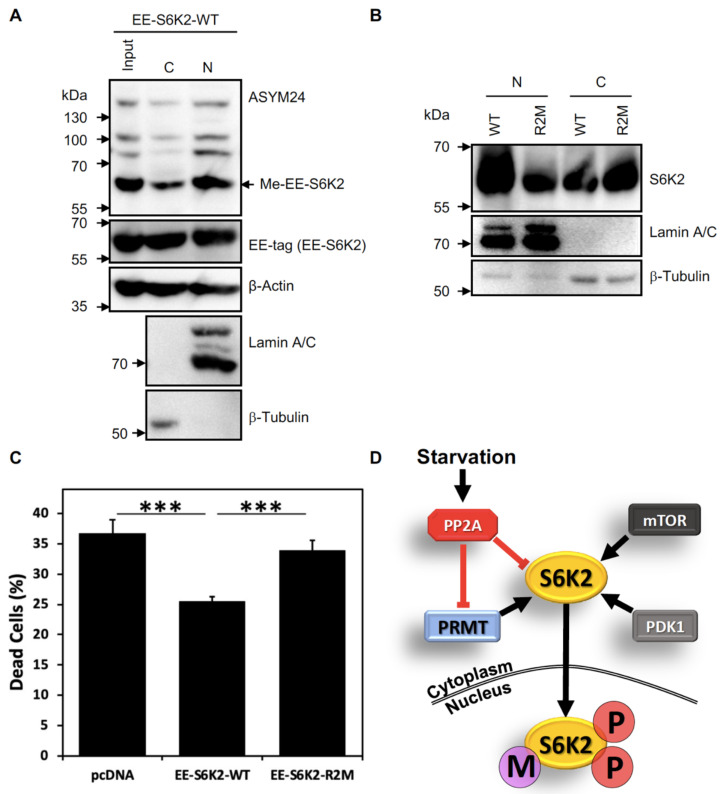
Methylation of S6K2 modulates its subcellular localisation and protects cells from starvation-induced cell death. (**A**) Methylated EE-S6K2 is predominantly localised in the nucleus in exponentially growing HEK293 cells. T-Rex-HEK293 cells stably expressing EE-S6K2 (WT) were fractionated for cytoplasmic (C) and nuclear (N) pools. Whole lysates (input) and fractions were analysed by SDS-PAGE/immunoblotting using the indicated primary antibodies. The purity of the fractions was evaluated by blotting with anti-lamin A/C and anti-β-tubulin antibodies. (**B**) The R2M mutant is localised in the cytosol of HEK293 cells. T-Rex-HEK293 cells stably expressing EE-S6K2 (WT) or the R2M mutant were fractionated for cytoplasmic (C) and nuclear (N) pools. The fractions were analysed by SDS-PAGE/immunoblotting using the indicated primary antibodies. (**C**) Wild-type S6K2 (WT), but not the R2M mutant, rescues cells from starvation-induced cell death. HEK293 stably expressing EE-S6K2-WT, EE-S6K2-R2M, or empty vector (pcDNA) were incubated in serum-free media for 24 h. Cells were collected and the proportion of dead cells was assessed using Trypan blue exclusion. The data represent the mean ± SEM of *n* = 12 (*** *p* < 0.001, Student *t*-test). (**D**) Proposed model for the regulation of arginine methylation of S6K2. PRMT, protein arginine methyltransferase; PP2A, protein phosphatase 2A; mTOR, mammalian target of rapamycin: PDK1, 3-Phosphoinositide-dependent kinase 1; P, phosphorylation; M, arginine methylation. (**A**–**C**) All data shown are representative of *n* = 3 biological repeats.

In view of the above, we wondered if methylation could modify the cellular localisation of S6K2. To test this, we performed subcellular fractionation of HEK293 cells expressing wild-type or arginine-mutant S6K2. This revealed that methylated S6K2 was present in the nuclear fraction (Figure 4A), while the R2M mutant appeared mostly cytoplasmic (Figure 4B). This, together with our finding that serum and FGF2 stimulation increases S6K2 methylation (Figure 2D,F), agrees with prior knowledge that S6K2 localises to the cytoplasm of serum-starved cells and translocates to the nucleus upon growth factor stimulation [5]. PRMT-mediated methylation has previously been shown to impact the nucleocytoplasmic distribution of its targets, including Sam68, RNA helicase A, and hnRNPA [9,22,36]. This can be associated with changes in phosphorylation status, as in the case of STAT6 [37], STAT1 [38], and the Npl3p RNA-binding protein [39]. Hence, methylation may influence or cooperate with other PTMs to regulate protein localisation and activity. In support of this, PP2A, the main phosphatase for S6Ks, is activated by amino acid deprivation [40], a condition that occurs under serum starvation that we found to demethylate S6K2. PP2A is known to bind PRMT1, inhibiting its methyltransferase activity [41]. Hence, activation of PP2A under serum starvation could inactivate S6K2 and simultaneously inhibit the dimethylation of this kinase, excluding it from the nucleus (Figure 4D).

We previously demonstrated that S6K2 regulates cell survival through phosphorylation of the nuclear protein hnRNPA1 [2,14] and showed increased nuclear localisation of this kinase in breast cancer [7] where it drives pro-survival signaling [14]. We therefore hypothesised that arginine methylation of S6K2 may promote cell survival. HEK293 cells expressing either the wild-type or R2M mutant S6K2 were serum-starved for 24 h and their cell viability was evaluated. As shown in Figure 4C, cells expressing R2M-S6K2 exhibited significantly (*p* < 0.001) increased cell death as compared to cells expressing the wild-type kinase. Taken together, our data suggest that the pro-survival function of S6K2 depends on its dimethylation and positively corelates with its nuclear localisation.

Our findings contribute to our understanding of S6K2 activation and its biological function. Indeed, PKCε-mediated phosphorylation of receptor-interacting protein 140 (RIP140) stimulated its arginine methylation and subsequent cytoplasmic localisation [42]. Interestingly, PKCε interacts with S6K2 and sequesters it in the cytoplasm through phosphorylation of Ser-486, which inhibits the NLS of this kinase [5]. In addition, interaction with PKCε is essential for the pro-survival activity of S6K2 in SCLC [2]. Hence, PKCε may regulate S6K2′s function through the coordination of phosphorylation and methylation events. In addition, C-terminal phosphorylation by ERK is essential for relieving the autoinhibition of S6K2 [3] and we reported that FGF-2 activates S6K2 in SCLC cells through a MEK-dependent pathway [2,43]. Here again, while we show that the kinase activity of S6K2 is dispensable for binding to PRMTs (Figure 1D), this phosphorylation event may somehow crosstalk with methylation of this kinase, a hypothesis that we will need to test in the future. Indeed, methylation can prevent nearby phosphorylation events, such as for the PRMT1-mediated Arg-248/250 dimethylation of FOXO1 which blocks its Ser-253 phosphorylation by AKT, leading to its nuclear accumulation and the subsequent induction of apoptosis [44]. Hence, the order in which PTMs occur on S6K2 may define the ultimate biological output obtained.

## 3. Materials and Methods

### 3.1. General Reagents

All of the general-purpose reagents were purchased from Thermo Fisher Scientific (Swindon, UK), Sigma-Aldrich (Burlington, MA, USA), Melford Laboratories Ltd. (Ipswich, UK), BDH AnalaR^®^ (Dorset, UK), and Fermentas (Waltham, MA, USA), or unless otherwise stated.

### 3.2. Reagents Used in the Cell Treatments

Rapamycin and LY294002 were obtained from LC Laboratories (Woburn, MA, USA). The InSolution^TM^ Sinefungin and Fibroblast growth factor-2 (FGF-2) were purchased from Calbiochem (San Diego, CA, USA). S-(5′-Adenosyl)-L-methionine chloride dihydrochloride (AdoMet) was purchased from New England BioLabs Inc. (Hitchin, UK). Sodium arsenite was purchased from Sigma-Aldrich (Burlington, MA, USA). The concentration used for each reagent is stated in the text or figure as necessary.

### 3.3. Expression Constructs and Recombinant Proteins

The construction of the pcDNA3.1/EE-p54-S6K2 expression plasmid has been previously described [1]. The pcDNA3.1/EE-p54-S6K2 mutants (R475M, R477M, R479P, R475M/R477M (R2M), and R475M/R477M/R479M (R3M)) were made using a QuickChange site-directed mutagenesis kit (Stratagene, La Jolla, CA, USA) with pcDNA3.1/EE-p54-S6K2 as the template. Other truncated mutants (deletion of 5 or 9 amino acids, Δ5 and Δ9, respectively) of p54-S6K2 were amplified by PCR using human S6K2 cDNA as a template. The amplicons were cloned into the BamH1/EcoR1 sites of the pcDNA3.1 plasmid (Invitrogen, Carlsbad, CA, USA) in frame with the N-terminal EE-tag (MEFMPME) [45].

The pGEX-2T/GST-PRMT1, pGEX-2T/GST-PRMT3, and pGEX-2T/GST-PRMT6 were kindly provided by Dr. Andrew Bannister and Dr. Tony Kouzarides (The Gurdon Institute, University of Cambridge, UK). The pcDNA3.1/myc-PRMT6 construct was kindly provided by Dr. Taras Valovka (Institute of Biochemistry, University of Innsbruck, Austria). All constructs were verified by restriction digestion and DNA direct sequencing. As described previously, His-S6K2 was purified from Sf9 insect cells and the 80S ribosomes were purified from a rat liver [5,46].

### 3.4. Antibodies

Polyclonal antibodies towards S6K2 and the monoclonal antibody to the EE-tag were described previously [5]. The anti-PRMT1 (07-404), anti-PRMT3 (07-256), and anti-asymmetric dimethylarginine (ASYM24) (07-414) antibodies were purchased from Upstate Biotechnology, Inc. (Boston, MA, USA). The anti-PRMT6 antibody (IMG-506) was purchased from Imgenex (Bhubaneswar, India). The anti-phospho-rpS6 (Ser240/244) (2215), anti-phospho-p70 S6K (Thr389) (p-T412 S6K) (9205), and anti-Lamin A/C (2032) were purchased from Cell Signalling Technology (Danvers, MA, USA). The anti-β-tubulin (H-235) antibodies were purchased from Santa Cruz Biotechnology Inc. (Dallas, TX, USA), and the anti-β-actin (AC15) antibodies were obtained from Sigma-Aldrich (Burlington, MA, USA). The Anti-Mono/DiMethyl Arginine (MMA/DMA) antibody [7E6] (ab412) was obtained from Abcam (Boston, MA, USA). The horseradish peroxidase (HRP)-conjugated anti-mouse (W4021) and anti-rabbit HRP (W4011) antibodies were purchased from Promega Corporation (Madison, WI, USA). The monoclonal antibody to the EE-tag was a gift from Dr. Julian Downward (Oncogene Biology Laboratory, The Francis Crick Institute, London, UK). The monoclonal antibody to the Myc-tag was generated at Ivan Gout’s laboratory.

### 3.5. Cell Culture

The HEK293 and SCLC cell lines were obtained from the American Type Culture Collection (ATCC; Manassas, VA, USA) and maintained as per the instructions of the supplier (humidified atmosphere, 10% CO_2_, 37 °C). The HEK293 cells were grown in DMEM while the SCLC cells were grown in RPMI 1640, supplemented with 10% heat-inactivated FBS (Hyclone; Logan, UT, USA), 2 mM L-glutamine, 50 U/mL penicillin, and 0.25 μg/mL streptomycin. The general cell culture reagents were acquired from PAA Laboratories GmbH (Cölbe, Germany).

### 3.6. Establishing Tetracycline-Inducible p54-S6K2 Cell Lines

Tetracycline-inducible p54-S6K2 cell lines were generated using the Tetracycline-Regulated Expression System for mammalian cells (T-Rex System; Invitrogen, Carlsbad, CA, USA) as described previously [47]. Briefly, the cDNA sequences of p54-S6K2 or that of the mutants, with the N-terminal EE-tag, were cloned into a pcDNA4/TO-inducible vector and transfected, using ExGen 500 transfection reagent (Fermentas; Waltham, MA, USA), in T-Rex-HEK293 for 48 h. The transfected cells were selected in complete DMEM supplemented with 5 μg/mL blasticidin and 100 μg/mL zeocin (Sigma-Aldrich; Burlington, MA, USA). The cells were screened for tetracycline-regulated protein expression. The cells were maintained in complete DMEM supplemented with 1 μg/mL blasticidin. To induce protein expression, the cells were treated with 1 μg/mL tetracycline (Sigma-Aldrich; Burlington, MA, USA) and incubated for 24 h at 37 °C prior to experiments. 

### 3.7. DNA Transfections

For the DNA transfection studies, the cells were seeded at 5 × 10^6^ cells per well in a 6-well plate and transfected using ExGen 500 transfection reagent (Fermentas; Waltham, MA, USA) as recommended by the manufacturer’s instructions. The cells were harvested and lysed for analysis by immunoblotting and immunoprecipitation after 24–48 h of transfection.

### 3.8. Starvation-Induced Cell Death Assay

T-Rex-HEK293 cells stably expressing EE-S6K2 (WT), the R2M mutant, or the empty vector (pcDNA3.1) were plated in 12-well plates (2 × 10^4^ cells/well) and cell death was induced by serum starvation for 24 h. The percentage of dead cells was measured by the method of trypan blue exclusion [2].

### 3.9. Cell Lysis and Subcellular Fractionation

The cultured cells were rinsed once with ice-cold phosphate-buffered solution (PBS) and scraped into cell lysis buffer (20 mM Tris-HCl [pH 7.5], 50 mM NaF, 150 mM NaCl, 5 mM EDTA [pH 8.0], 1% TritonX-100, protease inhibitors mix (Roche; Basel, Switzerland)). After incubation on ice for 30 min, whole-cell lysates were centrifuged (10,000× *g*, 30 min, 4 °C) to pellet cellular debris. Protein concentrations in the cleared lysates were measured using the Bradford assay. A cytoplasmic fraction was prepared by suspending the cultured cells in ice-cold hypotonic buffer (20 mM HEPES [pH 7.9], 0.5 mM DTT) supplemented with a protease inhibitor cocktail for 15 min. This was followed by the addition of 40 μL/mL 10% Nonidet P-40 (NP-40) and 10 sec vortexing. After centrifugation (800× *g*, 5 min, 4 °C) to pellet the nuclei, the supernatant was subjected to centrifugation (10,000× *g*, 15 min, 4 °C) to collect the cytoplasmic fraction. To generate a nuclear fraction, nuclei were collected and washed once with hypotonic buffer supplemented with 240 μL/mL 10% NP-40 (800 g, 5 min, 4 °C). This was followed by a second wash in a hypotonic buffer alone. Nuclear pellets were lysed in ice-cold cell lysis buffer (20 mM Tris-HCl [pH 7.5], 150 mM NaCl, 50 mM NaF, 5 mM EDTA [pH 8.0], 1% Triton X-100, and a protease inhibitors cocktail). After incubation for 30 min at 4 °C, the nuclear fractions were separated from the insoluble nuclear matrix material by centrifugation at 10,000× *g* for 30 min at 4 °C.

### 3.10. Immunoprecipitation

Equal amounts of total protein from the generated whole-cell lysates or fractions were incubated with the specific antibody and 50% Protein G Sepharose suspension for 3 h or overnight on a rotating wheel at 4 °C. The beads were washed three times in ice-cold lysis buffer (450 g, 5 min, 4 °C), prior to the elution of bound immune complexes by boiling for 5 min in 2X Laemmli sample buffer. The eluted immune complexes were subjected to SDS-PAGE and analysed by immunoblotting using specific antibodies.

### 3.11. Immunoblotting

The SDS-PAGE-separated proteins were transferred onto 0.45 μm pore-size PVDF membranes (Millipore; Burlington, MA, USA) using Trans-Blot (Bio-Rad; Hercules, CA, USA). The membranes were blocked in 5% zero-fat-dried milk in Tris-buffered saline containing 0.1% Tween 20 prior to the incubation in primary antibodies for 1–3 h at room temperature or overnight at 4 °C. The membranes were subjected to several washes in Tris-buffered saline containing 0.1% Tween 20 (TBST) to remove excess antibodies. The immunoreactive proteins were detected by incubation with a species-specific horseradish peroxidase-conjugated secondary antibody for 1 h at room temperature. The immune complexes were detected with the Amersham Enhanced ChemiLuminescence system (Amersham Biosciences; Piscataway, NJ, USA) prior to scanning using the Fluor-S Max MultiImager system (Bio-Rad; Hercules, CA, USA).

### 3.12. Expression of GST-Fusion Proteins

For the expression of the GST-fusion proteins (GST-PRMT1, GST-PRMT3, and GST-PRMT6) in the bacteria, the fusion proteins were expressed in BL21 (DE3) cells. Expression was carried out at 25 °C for 3 h in the presence of 0.5 mM IPTG. The bacteria were centrifuged at 5000 rpm at 4 °C for 20 min. prior to being washed in ice-cold PBS and undergoing lysis in lysis buffer (20 mM Tris-HCl [pH 7.5], 50 mM NaF, 150 mM NaCl, 5 mM EDTA pH 8.0, 1% Triton, and protease inhibitor mixture) for 30 min on ice. Mild sonication on ice was carried out for the lysates prior to centrifugation for 30 min at 18,000 rpm at 4 °C. Glutathione-Sepharose 4B beads (Amersham Biosciences; Piscataway, NJ, USA) were used to batch-purify the GST-fusion proteins from the supernatant at 4 °C for 2 h. The beads were washed several times in ice-cold lysis buffer at 4 °C. GST-fusion proteins were either retained on the beads or eluted by competition with 20 mM glutathione in 100 mM Tris-HCl [pH 8.0] and 150 mM NaCl. The eluted fusion proteins were subjected to dialysis against dialysis buffer 1 (50 mM Tris-HCl [pH 7.5], 150 mM NaCl, and 1 mM DTT) overnight at 4 °C, and then against dialysis buffer 2 (buffer 1 plus 50% glycerol) for 3 h at 4 °C.

### 3.13. GST Pull-Down Assay

GST-PRMT1, GST-PRMT3, GST-PRMT6, or GST alone coupled to Glutathione-Sepharose 4B beads (Amersham Biosciences; Piscataway, NJ, USA) were incubated with recombinant His-S6K2 in binding buffer (20 mM Tris-HCl [pH 7.5], 150 mM NaCl, and 1% Triton, 1%BSA, 0.5% NP-40) for 3 h on a rotating wheel at 4 °C. The beads were then pelleted by centrifugation at 800× *g* for 2 min and washed in binding buffer four times. The complexes were boiled in 2X Laemmli sample buffer for SDS-PAGE analysis.

### 3.14. In Vitro Methylation Assay

The reaction of the in vitro methylation assay was carried out using recombinant GST-PRMTs and His-S6K2, the methyl donor [^3^H]AdoMet (3 μCi) (Amersham Biosciences; Piscataway, NJ, USA), and in the presence or absence of the methylation inhibitor InSolution^TM^ Sinefungin (500 μM) (Calbiochem; San Diego, CA, USA). The reactions were typically performed in 40 μL of 50 mM sodium phosphate buffer (pH 7.3) at 30 °C for 3 h with gentle shaking. The reaction products were resolved by SDS-PAGE. The gels were stained with Coomassie brilliant blue and destained overnight in 10% (*v*/*v*) methanol and 5% (*v*/*v*) acetic acid prior to the use of EN^3^HANCE (Amersham Biosciences; Piscataway, NJ, USA) for 1 h. To precipitate the fluor in the gel, cold water was added with gentle agitation for 30 min. The gels were dried and exposed to Fuji medical X-ray film for 48 h at −70 °C and the labelled proteins were visualised by fluorography.

### 3.15. In Vitro Kinase Assay

HEK293 cells were transfected with Myc-PRMT6 and EE-S6K2 expression plasmids. The cell extracts were subjected to immunoprecipitation with either anti-EE-tag or anti-Myc-tag antibodies following treatment for 30 min with the signal transduction inhibitors: 100 nM Rapamycin, 50 µM LY294002, or DMSO (vehicle). The immune complexes were washed three times with lysis buffer followed by a single wash with kinase assay buffer (50 mM HEPES [pH 7.5], 10 mM MgCl_2_, 1 mM dithiothreitol, 10 mM β-glycerophosphate). The kinase reaction was initiated in 25 μL of kinase assay buffer supplemented with 1 μM protein kinase A inhibitor (Calbiochem; San Diego, CA, USA), 50 μM ATP, 5 μCi of [γ-^32^P] ATP (Amersham Biosciences; Piscataway, NJ, USA), and 20 μg of 80S ribosomes isolated from a rat liver as a substrate (source of S6 protein (rpS6)) [5,46]. The reaction was carried out at 30 °C for 30 min and terminated by the addition of the 5X SDS-PAGE sample buffer and boiling for 10 min. The proteins were resolved by SDS-PAGE. The amount of [γ-^32^P] incorporated into the proteins was assessed by a Fujifilm FLA-2000 phosphoimager apparatus (Bio-Rad; Hercules, CA, USA). Control IP was achieved by incubating lysates with protein A-Sepharose beads.

## 4. Conclusions

We identified the Arg-475/477 dimethylation of S6K2, a new PTM regulating the subcellular localisation and biological activity of this kinase. Further research now needs to be conducted on how this coordinates with other signalling events and PTMs of this kinase and its relevance to cancer progression and associated therapeutic approaches.

## Data Availability

The data generated during and/or analysed during the current study are available from the corresponding author upon reasonable request.
